# Clinical Notes

**Published:** 1873-05-01

**Authors:** F. K. Bailey

**Affiliations:** Knoxville, Tenn.


### CLINICAL NOTES.

BY F. K. BAILEY, M.D., KNOXVILLE, TENN.

### DROPSY FROM DISEASE OF LIVER AND KIDNEYS.

January 24, 1873. Called to visit a woman about thirty-two; unmixed African; married; two children, the youngest about two years old.

I learned the following imperfect history. In July or August last she became pregnant, and during the autumn began to feel severe pain in the pit of the stomach, aggravated on evacuating the rectum.

Says her feet began to swell before cold weather set in, and a cough also troubled her. About 10th January she miscarried, at about seven months.

When I first saw her she was coughing and expectorating bloody sputa, and at times spitting out clear blood, of a dark hue. The feet and legs œdematous and cold. Hands and forearms also considerably swollen, and the face full. The pulse could scarcely be felt, on account of the effusion in the wrists; and the action of the heart was labored and irregular. Indications of pericardial effusion, with obstruction in the right side of the heart. Urine not scanty, but red and acid. Complains of pain in the pit of the stomach, extending towards the right side, aggravated on voiding the bowels. Tongue coated thick with a whitish fur; intense thirst for cold drinks. Sometimes craving for solid food.

Prescribed pulv. Doveri in six grain doses, with leptandrin two grains, and an expectorant syrup composed of ipecac and sang. canadensis.

The symptoms continued unchanged from day to day for ten days or more, when the pain in the stomach was less, the bowels became more regular and natural. Slight diminution in the œdema, and less blood in sputa. Gave Doveri powders as called for, and leptandrin, to keep up action of the bowels.

Towards the last of February there was an aggravation of symptoms. Œdema extended to the body, and upon the legs frequent rupture of the cuticle left openings, from which escaped a great amount of serum. I also gave fifteen drops tinct. mur. iron in a teaspoonful of a saturated solution of potash four times a day.

On examination of the urine, it was found to be highly albuminous, and epithelial casts also very obvious under the microscope. The expectoration was bloody during nearly the whole period of my attendance. About the first of March, for a few days, there was a cessation of the cough and expectoration, while at the same time there commenced frequent painful evacuations from the bowels, of a muco-purulent character. These discharges appeared to be associated with the pain in the pit of the stomach. During the last weeks of the case there was soreness and pain on pressure on the hepatic region, and there has unquestionably been some organic disease of the liver for some time.

Since March 1st there has been a failure in strength, with but little rest at night. During a part of the whole period embraced in this history the pâtient was unable to lie down at all. The pulse ranged from 110 to 120, and the respiration 30 or more. Most of the time it has been very difficult to determine the cardiac sounds; but there appeared to be no valvular lesion, except in the mitral. The woman lived until the 12th, and for forty-eight hours was constantly complaining of pain in the right side. She was a person of more than ordinary intelligence for one of her class, and her previous habits had been good.

The disease had been developing for some months, and was slowly but surely advancing to a fatal issue during each day of its continuance. There is a painful interest attached to such cases, where we can with but little difficulty decide upon a diagnosis, our prognosis equally easy, and the treatment but palliative in its effects.

### DROPSY FROM CARDIAC DISEASE.

February 7, 1873: R. D., single; aged twenty-three; light complexion; but, according to his mother's account, "not very strong. For three or four years past he has been irregular in his habits, spending much of his time drinking, lying out in stormy weather, and often in the calaboose.

Besides, he has been very licentious, and all his conduct calculated to induce disease. January 18th ult., he was out all day and to a late hour at night, it being the annual city election. On the 19th he was taken with severe pain in the chest, attended with fever.

He was visited three or four times by one of our physicians, who ceased his attendance for some reason, and I was requested to see him on the day above stated. I found him sitting in a chair, breathing rapidly and laboriously. The feet and legs were œdematous, the face full, and the hands somewhat bloated. The pulse was 100, but irregular. There was dullness over an extended area in the cardiac region, with greatly increased impulse, as seen externally.

On applying the ear, there was friction sound, together with a splashing, in the whole region of the heart, and continued towards the left clavicle. It was difficult to fully determine whether there was regurgitation into the left ventricle, but it was probable. Circulation much obstructed in the right side of the heart, as indicated by dyspnœa and a constant spitting of blood. Of this latter accompaniment there is no cessation for a moment. Every time he coughs or expectorates, blood is passed out, and mostly dark colored. His mouth was most of the time filled with blood, rendering his sleep imperfect at night, even if otherwise possible to obtain rest.

Sometimes he could lie down at night, but never on the left side. Again, and especially as the disease advanced, he could not lie down at all. The urine was rather copious, but albuminous. No tubular casts; bowels confined; some appetite; thirst for cold drinks. I prescribed iod. potassi in doses of seven and a half grains three times daily, with tinct. digitalis and actea racemosa. After about a week, alternated the above with chlorate potass and tinct. muriate iron. Having not the least idea of accomplishing anything in the case, but a palliation of symptoms, the above was continued, with some mild laxative. About the 10th, the cuticle gave way in the legs and feet, and the serum escaped in great quantities. From day to day there was a failure of strength, and an increase of the amount of blood from the lungs.

On the morning of the 21st, at four o'clock, I was called, and found him sinking. The pulse very weak and rapid; respiration much labored; and within thirty minutes blood began to pour from his mouth and nostrils in full streams, and life was extinct. There was no *post mortem;* and, from various causes, it was not possible to ascertain what were the first symptoms. It is probable that from exposure to cold and wet, inflammation occurred in the pericardium, and perhaps in the endocardium. The case was interesting from the violent character of the symptoms, especially in the heart. There might have been some cardiac trouble for years, as his mother said he was not a stout boy.

I am led to believe that, other things being equal, the subjects of cardiac disease are apt to become intemperate in drinking. The unpleasant sinking and feeling of exhaustion accompanying disease of the heart may induce the desire for stimulants; and, again, the habits and associations of drunkards tend in turn to aggravate the disease thus perchance produced.

